# Chronic kidney disease, preoperative use of antispasmodics and lower resected prostate volume ratios are risk factors for postoperative use of adrenergic Alpha-blockers and antispasmodics

**DOI:** 10.1371/journal.pone.0282745

**Published:** 2023-03-09

**Authors:** Chen-Hsun Hsueh, Li-Wen Chang, Kun-Yuan Chiu, Sheng-Chun Hung, Jun-Peng Chen, Jian-Ri Li

**Affiliations:** 1 Department of Urology, Taichung Veterans General Hospital, Taichung, Taiwan; 2 School of Medicine, National Chung Hsing University, Taichung, Taiwan; 3 Department of Medical Research, Taichung Veterans General Hospital, Taichung, Taiwan; 4 Department of Intensive Care, Division of Surgical Intensive Care Unit, Taichung Veterans General Hospital, Taichung, Taiwan; 5 Department of Nursing, Hungkuang University, Taichung, Taiwan; King Abdulaziz University, UNITED STATES

## Abstract

**Objectives:**

Transurethral resection of prostate (TURP) and laser prostate surgery are common surgeries for benign prostate hyperplasia (BPH). We conducted an investigation using hospital database to evaluate the clinical factors associated with post-operative usage of alpha-blockers and antispasmodics.

**Methods:**

This study was conducted using retrospective clinical data from the hospital database, which contained newly diagnosed BPH patients between January 2007 and December 2012 who subsequently received prostate surgery. The study end-point was the use of alpha-blockers or antispasmodics for at least 3 months duration after 1 month of surgery. The exclusion criteria was prostate cancer diagnosed before or after the surgery, recent transurethral surgeries, history of open prostatectomy, and history of spinal cord injury. Clinical parameters, including age, body mass index, preoperative prostate specific antigen value, comorbidities, preoperative usage of alpha-blockers, anstispasmodics and 5-alpha reductase inhibitors, surgical methods, resected prostate volume ratios, and preoperative urine flow test results, were evaluated.

**Results:**

A total of 250 patients receiving prostate surgery in the database and confirmed pathologically benign were included. There was significant association between chronic kidney disease (CKD) and the usage of alpha-blockers after prostate surgery (OR = 1.93, 95% CI 1.04–3.56, p = 0.036). Postoperative antispasmodics usage was significantly associated with preoperative usage of antispasmodics (OR = 2.33, 95% CI 1.02–5.36, p = 0.046) and resected prostate volume ratio (OR = 0.12, 95% CI 0.02–0.63, p = 0.013).

**Conclusions:**

BPH patients with underlying CKD were more likely to require alpha-blockers after surgery. In the meantime, BPH patients who required antispasmodics before surgery and who received lower prostate volume resection ratio were more liable to antispasmodics after prostate surgery.

## Introduction

Benign prostate hyperplasia (BPH) is a common problem in elder patients, with a prevalence of over 50% in male population aged over 50 years [1]. BPH may lead to prostate enlargement that obstructs bladder neck, which further causes lower urinary tract symptoms (LUTS). Symptomatic LUTS can be manifested as storage symptoms and voiding symptoms, and may result in decreased quality of life as well as various complications [2]. Treatment of BPH-induced LUTS is indicated to relieve symptoms and prevent complications. The treatment begins with lifestyle modification and medical treatment, and may proceed to surgical intervention if initial treatments fail. Most patients present with general improved LUTS symptoms after surgery [3,4]. However, some patients still require medical treatment, especially adrenergic alpha-blockers and antispasmodics, after surgery due to recurrent BPH or persistent LUTS symptoms [5,6]. Obviously, there may be some different characteristics in this patient group that hinder the effects of surgical interventions. Further investigation of this patient group is essential to provide better treatment suggestion in advance. In this study, we aim to identify BPH patients who require adrenergic alpha-blockers and antispasmodics for at least three months after receiving surgery using preoperative and perioperative data.

## Methods

### Database

Our database was a web-based system that recorded clinical information of patients receiving medical care in Taichung Veterans General Hospital (VGHTC) since January 2000. It included diagnosis, operation notes, results of examinations from outpatient departments, emergency departments and hospitalizations, as well as records of prescribed medicine. The patient data was de-identified before our analysis for privacy concern.

### Patient selection and treatments

Patients who were newly diagnosed BPH between January 2007 and December 2012, and subsequently received prostate surgery were included. The ICD code for BPH was ICD-9-CM 600. The exclusion criteria included prostate cancer diagnosed before or after surgery, transurethral incision or resection within a year before surgery, history of open prostatectomy, and history of spinal cord injury. History of prostate cancer was identified by medical records, preoperative transurethral biopsy results, and biopsy retrieved peri-operatively. All patients included were followed up to December 2021 for last hospital visit or death.

### Study process

This study was conducted using retrospective clinical data from VGHTC database, and was approved by institutional review board with No. CE21221A. The study endpoints included the usage of adrenergic alpha-blockers and antispasmodics after surgery for at least three months. Most patients returned back to the outpatient clinic one week after discharge and were followed up every three months afterward. The cut-off of minimum three months was identical to previous study, and was determined based on the fact that some patients may require short-term medications for LUTS [6]. The adrenergic alpha-blockers and antispasmodics prescribed within a month postoperatively were neglected since these medicines prescribed then may be for operation-related symptom relief. Data collected were classified into preoperative data and perioperative data. Preoperative data included age, body mass index (BMI), prostate specific antigen (PSA), comorbidities (hypertension, ICD9 401–405, ICD10 I10-I16; diabetes mellitus [DM], ICD9 250, ICD 10 E08-E13; ischemic heart disease, ICD9 410–414, ICD10 I20-I25; cerebrovascular disease, ICD9 430–438, ICD I60-I69; hyperlipidemia, ICD9 272, ICD10 E78; chronic obstructive pulmonary disease [COPD], ICD9 490–496, ICD10 J40-J47; peripheral vascular disease, ICD9 440–449, ICD10 I70-I79; chronic kidney disease [CKD], ICD9 585, ICD10 N18; sleep disorder, ICD9 327, ICD10 G47; gout ICD9 274, ICD10, M10), history of prostate surgery, urodynamic testing results, and the usage of adrenergic alpha-blockers, 5-alpha reductase inhibitors (5-ARIs), and antispasmodics before surgery. The age and BMI data was collected at time of operation. PSA level was collected within a year before surgery. Urodynamic testing included maximum flow rate, average flow rate, voided volume and post-void residual volume, and was performed within a year preoperatively. The adrenergic alpha-blockers, 5-ARIs and antispasmodics were coded using anatomical therapeutic chemical classification. The adrenergic alpha-blockers and 5-ARIs included Doxazosin 2mg and 4mg (C02CA04), Alfuzosin 10mg (G04CA01), Tamsulosin 0.2mg and 0.4mg (G04CA02), Terazosin 1mg and 2mg (G04CA03), Silodosin 4mg (G04CA04), Tamsulosin plus Dutasteride 0.4mg/0.5mg (G04CA52), Dutasteride 0.5mg (G04CB02), Finasteride 5mg (G04CB01). The antispasmodics included Oxybutynin 5mg (G04BD04), Tolterodine 2mg and 4mg (G04BD07), Solifenacin 5mg (G04BD08), Trospium 10mg (G04BD09), and Mirabegron 25mg and 50mg (G04BD12). Usage of adrenergic alpha-blockers, 5-ARIs and antispasmodics preoperatively were only adopted if the usage duration last at least three months.

On the contrary, perioperative data included surgical methods and resected prostate volume to preoperative prostate volume ratios. Surgical methods contained TURP, which included monopolar TURP and bipolar TURP, and laser procedure, which included laser enucleation of prostate and photoselective vaporization. Resected prostate weight to preoperative prostate volume ratios were calculated using resected prostate weight, which was based on final histopathology weight, and preoperative prostate volume, which was measured by trans-abdominal ultrasound performed within a year before surgery.

### Statistical analysis

Data in this study were assessed using IBM SPSS version 22.0 (International Business Machines Corp, New York, USA). Logistic regression was performed to evaluate the impact of individual factor on postoperative medications. P < 0.05 was considered to demonstrate statistical significance, and all P values were 2-sided in this study.

## Results

There were 341 patients in our database that were newly diagnosed BPH within January 2007 to December 2012, and further received prostate surgery. Seventy patients with prostate cancer diagnosed before or after surgery were excluded. In addition, 16 patients receiving transurethral incision within a year before surgery, and 5 patients with history of open prostatectomy were also excluded. Eventually, there were 250 patients enrolled in this study. Seven patients (2.8%) were loss to follow up. The median follow-up duration was 5.3 years. The baseline characteristics were listed in Table 1. There were 146 (58.4%) patients with hypertension, 78 (31.2%) patients with DM, 59 (23.6%) patients with COPD, and 61 (24.4%) patients with CKD. Among all 61 CKD patients, 29 patients (47.5%) had DM. As for preoperative medical history, 56.8% patients had received adrenergic alpha-blockers for at least three months, while 22.4% patients had undergone 5-ARIs, 11.6% patients had undergone antispasmodics, and 11.2% patients had undergone a combination therapy of adrenergic alpha-blockers and antispasmodics. As for endpoints, 108 (43.2%) patients received postoperative adrenergic alpha-blockers, and 58 (23.2%) patients received postoperative antispasmodics. In addition, 36 (14.4%) patients received postoperative medications of both adrenergic alpha-blockers and antispasmodics. The median time to first use of postoperative adrenergic alpha-blockers was 2.28 months (IQR 1.36–10.52), and the median time to first use of postoperative antispasmodics was 1.90 months (IQR 1.27–4.88).

**Table 1:** Patient characteristics.

Characteristics	Patients (n = 250)
Age (year)	71.14 ± 9.26
Body mass index	24.83 ± 3.29
Prostate specific antigen level (ng/mL)	6.62 ± 7.36
Comorbidities	
Hypertension, n(%)	146 (58.4%)
Diabetes mellitus, n(%)	78 (31.2%)
Ischemic heart disease, n(%)	63 (25.2%)
Cerebrovascular disease, n(%)	59 (23.6%)
Hyperlipidemia, n(%)	67 (26.8%)
Chronic obstructive pulmonary disease, n(%)	59 (23.6%)
Peripheral vascular disease, n(%)	21 (8.4%)
Chronic kidney disease, n(%)	61 (24.4%)
Sleeping disorder, n(%)	12 (4.8%)
Gout, n(%)	32 (12.8%)
Medical history	
History of adrenergic alpha-blockers for over 3 months preoperatively	142 (56.8%)
History of 5-alpha reductase inhibitors for over 3 months preoperatively	56 (22.4%)
History of antispasmodics for over 3 months preoperatively	29 (11.6%)
History of combination therapy of adrenergic alpha-blockers and antispasmodics for over 3 months preoperatively	28 (11.2%)
History of prostate surgery	13 (5.2%)
Preoperative urodynamic study	
Maximum flow rate (ml/sec)	9.18 ± 4.65
Average flow rate (ml/sec)	3.83 ± 2.16
Post-void residual volume	104.65 ± 109.30
Voided volume	180.41 ± 110.97
Perioperative factors	
Surgical intervention with TURP	140 (56%)
Surgical intervention with laser procedure	110 (44%)
Resected prostate volume to preoperative prostate volume ratio	0.34 ± 0.20
Endpoints	
Patients receiving postoperative adrenergic alpha- blockers	108 (43.2%)
Patients receiving postoperative antispasmodics	58 (23.2%)
Patients receiving both adrenergic alpha-blockers and antispasmodics postoperatively	36 (14.4%)
Median time to first use of postoperative adrenergic alpha-blockers (months)	2.28 (IQR 1.36–10.52)
Median time to first use of postoperative antispasmodics (months)	1.90 (IQR 1.27–4.88)

Data show the number of patients, with percentage revealed in parenthesis, and the mean with standard deviation in each group.

In Table 2, we identified the following as risk factors for postoperative adrenergic alpha-blockers usage by univariate analysis: hypertension (OR 1.84, P = .021), COPD (OR 1.96, P = .025) and CKD (OR 2.34, P = .005). Multivariate analysis further disclosed the CKD (OR 1.93, P = .036) as the only independent risk factor for postoperative adrenergic alpha-blockers.

**Table 2:** Logistic regression analyzing factors that are associated with postoperative adrenergic alpha-blockers usage.

	Univariate			Multivariate			
	OR	95%CI	pvalue	OR	95%CI		pvalue
Age	1.00	(0.98–1.03)	0.767				
Body mass index	1.07	(0.99–1.15)	0.096				
Prostate specific antigen level	1.00	(0.97–1.04)	0.780				
Comorbidities							
Hypertension, n(%)	1.84	(1.09–3.09)	0.021*	1.54	(0.90–2.65)		0.114
Diabetes mellitus, n(%)	1.61	(0.94–2.76)	0.083				
Ischemic heart disease, n(%)	1.64	(0.92–2.92)	0.090				
Cerebrovascular disease, n(%)	1.64	(0.91–2.95)	0.099				
Hyperlipidemia, n(%)	1.19	(0.68–2.08)	0.554				
Chronic obstructive pulmonary disease, n(%)	1.96	(1.09–3.54)	0.025*	1.66	(0.90–3.06)		0.102
Peripheral vascular disease, n(%)	2.87	(1.12–7.39)	0.029				
Chronic kidney disease, n(%)	2.34	(1.30–4.22)	0.005**	1.93	(1.04–3.56)		0.036*
Sleeping disorder, n(%)	0.64	(0.19–2.20)	0.483				
Gout, n(%)	1.03	(0.49–2.17)	0.946				
Medical history							
History of adrenergic alpha-blockers for over 3 months preoperatively	1.56	(0.94–2.60)	0.087				
History of 5-alpha reductase inhibitors for over 3 months preoperatively	0.81	(0.44–1.49)	0.502				
History of antispasmodics for over 3 months preoperatively	1.26	(0.58–2.74)	0.558				
History of prostate surgery	1.13	(0.37–3.48)	0.825				
Preoperative urodynamic study							
Maximum flow rate	1.00	(0.95–1.05)	0.939				
Average flow rate	1.05	(0.93–1.17)	0.440				
Post-void residual volume	1.00	(1.00–1.00)	0.372				
Voided volume	1.00	(1.00–1.00)	0.311				
Perioperative factors							
Surgical intervention with laser procedure	0.79	(0.48–1.31)	0.366				
Resected prostate volume to preoperative prostate volume ratio	0.78	(0.22–2.71)	0.694				

Logistic regression was performed, and * indicates p<0.05, while ** indicates p<0.01.

In Table 3, univariate analysis revealed the following risk factors for postoperative antispasmodics usage: preoperative usage of antispasmodics (OR 2.69, P = .016) and surgical intervention with laser procedure (OR 1.97, P = .025). In addition, the resected prostate weight to preoperative prostate volume ratio (OR 0.08, P = .003) was disclosed as a protective factor against postoperative antispasmodic medications. Multivariate analysis further disclosed preoperative antispasmodics usage (OR 2.33, P = .046) as a risk factor for postoperative antispasmodic medications, whereas resected prostate weight to preoperative prostate volume ratio (OR 0.12, P = .013) was shown to be a protective factor.

**Table 3:** Logistic regression analyzing factors that are associated with postoperative antispasmodics.

	Univariate			Multivariate			
	OR	95%CI	pvalue	OR	95%CI		p value
Age	0.99	(0.96–1.03)	0.754				
Body mass index	1.04	(0.95–1.13)	0.431				
Prostate specific antigen level	0.98	(0.93–1.03)	0.384				
Comorbidities							
Hypertension, n(%)	1.34	(0.73–2.46)	0.343				
Diabetes mellitus, n(%)	1.34	(0.72–2.50)	0.349				
Ischemic heart disease, n(%)	0.93	(0.47–1.84)	0.832				
Cerebrovascular disease, n(%)	1.48	(0.76–2.87)	0.244				
Hyperlipidemia, n(%)	0.94	(0.48–1.83)	0.854				
Chronic obstructive pulmonary disease, n(%)	1.85	(0.97–3.54)	0.063				
Peripheral vascular disease, n(%)	1.04	(0.36–2.97)	0.945				
Chronic kidney disease, n(%)	1.94	(1.02–3.68)	0.043				
Sleeping disorder, n(%)	1.11	(0.29–4.24)	0.880				
Gout, n(%)	1.12	(0.47–2.65)	0.796				
Medical history							
History of adrenergic alpha-blockers for over 3 months preoperatively	1.46	(0.80–2.68)	0.221				
History of 5-alpha reductase inhibitors for over 3 months preoperatively	1.14	(0.57–2.27)	0.717				
History of antispasmodics for over 3 months preoperatively	2.69	(1.20–6.02)	0.016*	2.33	(1.02–5.36)		0.046*
History of prostate surgery	0.26	(0.03–2.07)	0.204				
Preoperative urodynamic study							
Maximum flow rate	1.03	(0.97–1.09)	0.379				
Average flow rate	1.04	(0.92–1.19)	0.524				
Post-void residual volume	1.00	(1.00–1.00)	0.375				
Voided volume	1.00	(1.00–1.00)	0.123				
Perioperative factors							
Surgical intervention with laser procedure	1.97	(1.09–3.57)	0.025*	1.83	(0.99–3.38)		0.054
Resected prostate volume to preoperative prostate volume ratio	0.08	(0.02–0.42)	0.003**	0.12	(0.02–0.63)		0.013*

Logistic regression was performed, and * indicates p<0.05, while ** indicates p<0.01.

**Fig 1:**
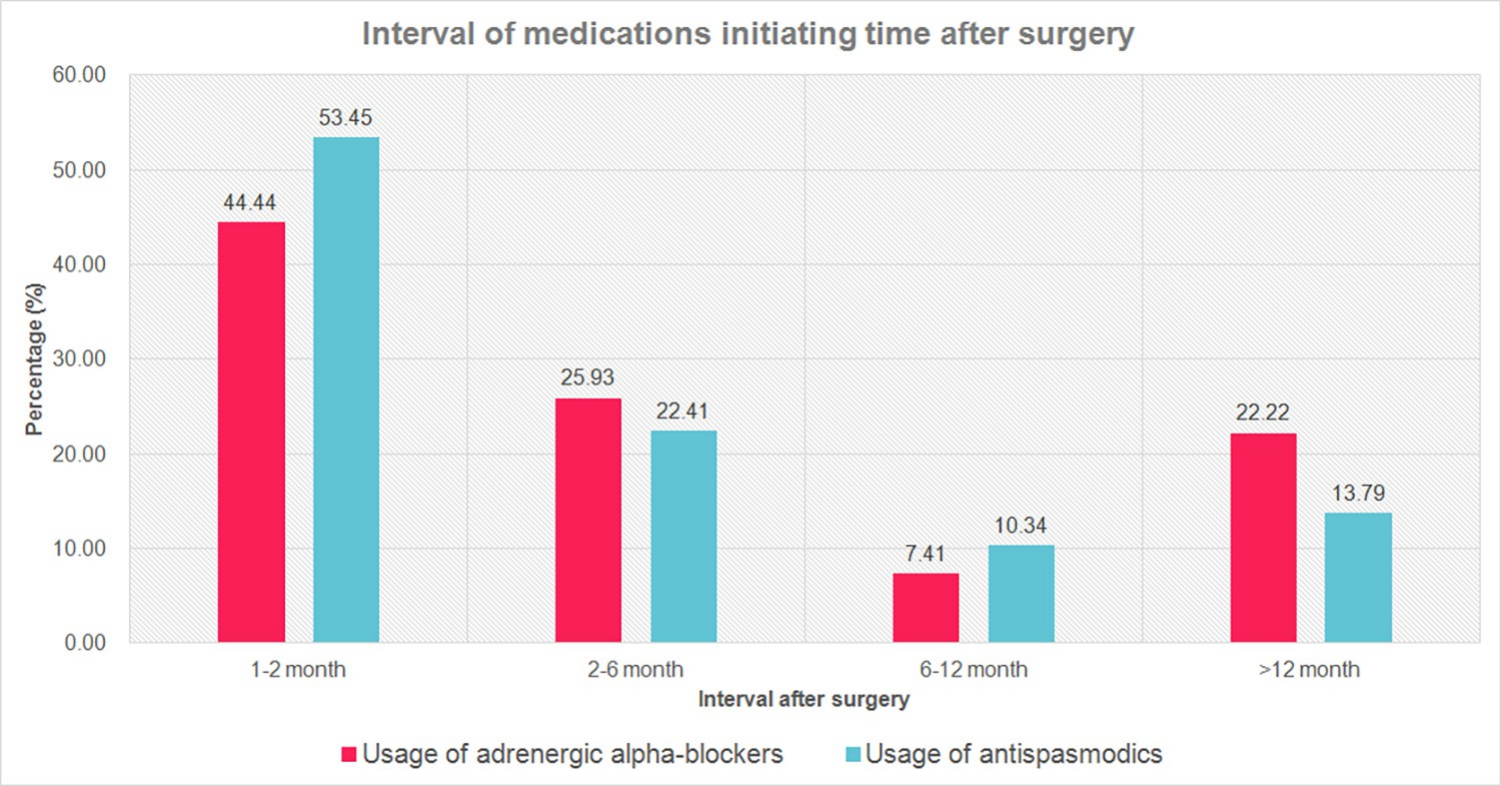
Interval of adrenergic alpha-blockers and medications for OAB initiating time after surgery.

**Fig 2:**
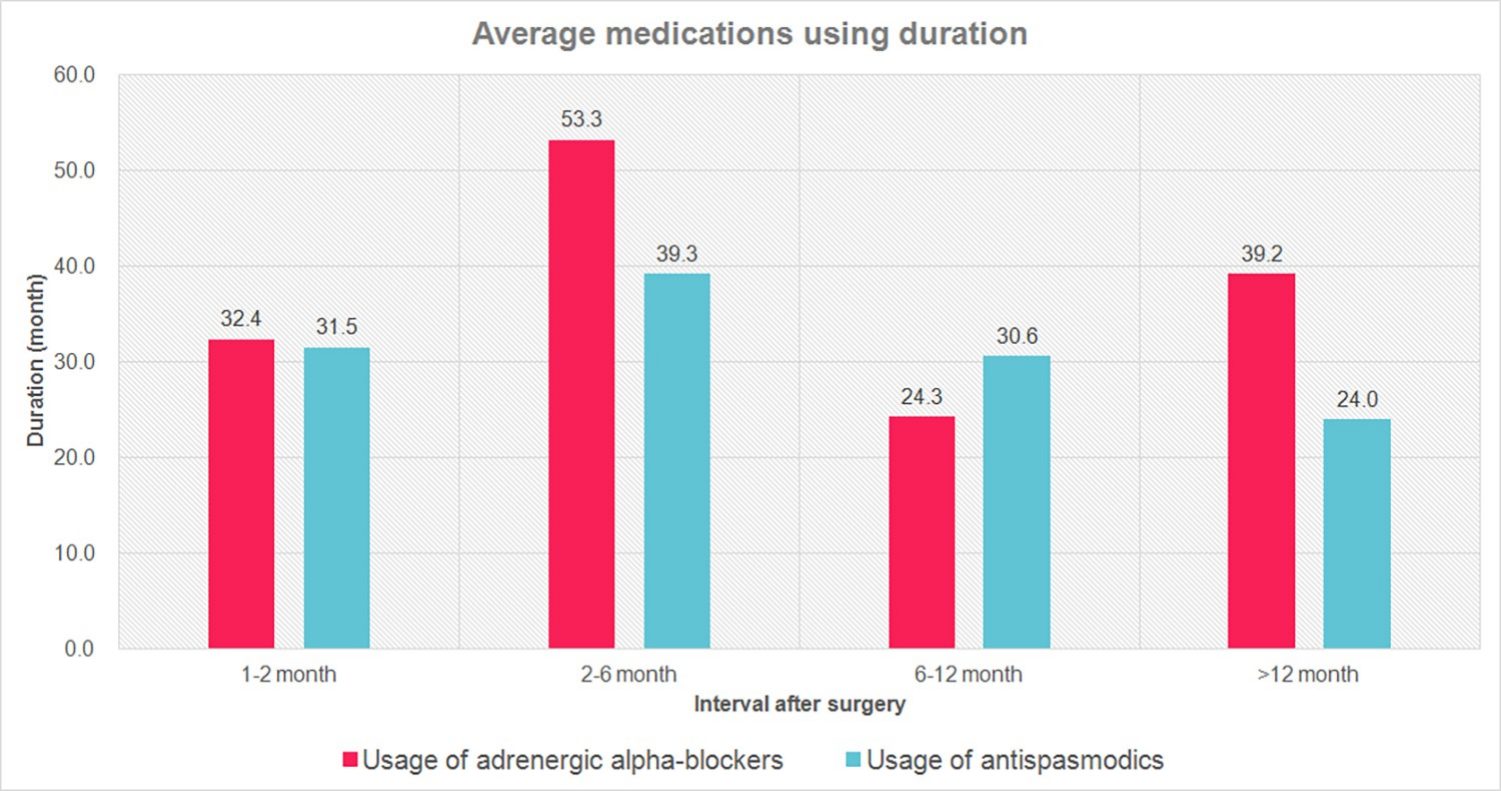
Average adrenergic alpha-blockers and antispasmodics using duration with respect to medicine initiating times.

The time to first use of adrenergic alpha-blockers and antispasmodics after surgery were recorded and demonstrated in Fig 1. The duration of medications usage with respect to the initiating time was demonstrated in Fig 2.

## Discussion

Our study showed several observational data which can assist clinical practices in BPH patients. First, the prostate surgery rate among newly diagnosed BPH patients was 4.88% (250/5122) in our database during a mean follow-up duration of 5.3 years. Second, we found that BPH patients having comorbid CKD were more liable to using adrenergic alpha-blockers after surgery. Third, patients undergoing antispasmodic medications for at least three months before surgery were more likely to initiate OAB medications postoperatively. Fourth, resected higher prostate volume ratio reduces the usage of antispasmodics postoperatively.

In BPH, the hyperplastic volume of prostate is mainly composed of stromal elements. Approximately half of the stromal elements were smooth muscle, which contracts when the alpha 1 adrenoceptors it contains are activated [7]. The contraction of prostatic smooth muscle blocks bladder outlet, and may further lead to bladder dysfunction, which results in LUTS symptoms [8,9]. Adrenergic alpha-blockers act by antagonizing those alpha 1 adrenoceptors, and further alleviates LUTS symptoms. In our study, CKD was associated with increased usage of postoperative adrenergic alpha-blockers usage. However, the underlying rationale remains unclear. Previous studies discovered association between LUTS symptoms and CKD [10,11]. It was postulated vascular diseases and DM that frequently accompanied CKD may cause bladder dysfunction, which further leads to LUTS symptoms [12]. Atherosclerosis may further result in inflammation and oxidative stress of the bladder that facilitates the development of detrusor overactivity. These comorbidities of CKD do not disappear after prostate surgery, and may altogether lead to persistent LUTS symptoms. This may explain why patients with CKD are more likely to undergo adrenergic alpha-blockers after surgery. Based on the clinical practice, the prescription of adrenergic alpha-blockers was based on either LUTS or patient’s preference. However, a recent study revealed that adrenergic alpha-blockers may be associated with higher risk for kidney disease progression in CKD patients, which should be aware in clinical conditions [13].

In order to recognize precise overactive bladder (OAB) patients, we used antispasmodics prescription to identify the target subjects instead of using ICD codes. OAB may be caused by BPH-induced bladder outlet obstruction (BOO). The coexistence of BPH-induced BOO and OAB increases the complexity for treatment [14]. Even worse, the OAB symptoms may persist after relief of BOO [9,15]. This is compatible with our study finding that 23.2% of patients who underwent prostate surgery receive medications for OAB postoperatively. Medications for OAB were antispasmodics, which include beta-3 adrenergic agonists, antimuscarinics, anticholinergics, and some tricyclic antidepressants that act to relax detrusor muscles [16,17]. In this study, we found patients who use antispasmodic medications preoperatively were more liable to antispasmodics usage after surgery. The finding was compatible to previous studies, which revealed that history of anticholinergics therapy was associated with continued medical therapy for LUTS after prostatic surgery [6,18–20]. Patients have to be informed the potential need of postoperative medications so as to avoid unnecessary expectations.

Univariate analysis showed association between surgical methods and postoperative antispasmodics usage, while multivariate analysis revealed no correlation. The surgical methods in this study contain TURP, which includes monopolar TURP and bipolar TURP, and laser procedure, which includes laser enucleation of prostate and photoselective vaporization. Previous studies suggested laser procedures were comparable to TURP in surgical and functional outcomes [21–23]. However, laser procedures were shown to be associated with higher postoperative usage of antispasmodic medications as compared to TURP in a study [18]. The difference between our study and previous studies is that our study included laser enucleation, while previous study did not. This put the effect of laser enucleation on postoperative antispasmodic medications usage into request for further investigation.

The lower resected prostate weight to preoperative volume ratio was found to be an independent risk factor for postoperative antispasmodic medications usage in our study. One study investigated the relationship between residual prostate volume and postoperative clinical outcomes using LUTS evaluation questionnaire and urinary flow rate, and negative correlation was shown [24]. Another study revealed weak relationship between resected prostate volume and early symptom relief after surgery [25]. The results of our studies were compatible with previous studies, but instead of using symptom improvement as evaluation metrics, we measured the usage of antispasmodic medications. International Prostate Symptom Score (IPSS) was considered an adequate tool to evaluate storage symptoms. However, the retrospective setting of this study and real-world practice showed massive missing data in this category. In addition, we also believe the use of antispasmodics provides a more practical information for urologists to evaluate whether patients may require postoperative medications or not during surgery.

We analyzed the time to first use of medications after surgery in this study, which showed most medications were initiated within a year postoperatively. The median time to first use of postoperative adrenergic alpha-blockers and antispasmodics were 2.28 months and 1.90 months respectively in our study. Previous study from Canada showed a median time to first use of adrenergic alpha-blockers and antispasmodics after TURP to be 1.25 years and 0.69 years respectively, which was longer than our study [20]. They excluded the prescription of medications within the first 90 days after TURP, which was longer than the interval of a month set in our study. This may explain the difference in time to first use of postoperative medications in our studies. In addition, medical availability between different countries may also be a cause of the difference. Previous studies proposed that prostatic surgery destroys prostatic and bladder neck urothelium and submucosal tissues, which may result in denervation of the afferent neurons. This further hinders the neurons from initiating detrusor contraction that causes OAB symptoms [26,27]. The proposed mechanism may explain why approximately half of the patients with OAB symptoms benefit from surgery in short term follow up [28,29]. However, it could not explain why some of the patients in our study still require medications 1–2 months after surgery, as the neurons responsible for OAB symptoms were damaged during surgery, and could not recover within that period. For those patients with persistent OAB symptoms after surgery, further investigation should be performed. Besides, it seemed that the number of patients needing post operative antispasmodic exceeded that in the pre-operative (23.2% vs 11.6%). This result was concordant with the trend presented by Campbell et al. in 2019 in which that storage problems were most difficult to be relieved by TURP. Furthermore, the utilization of antispasmodics after TURP increased over time. These might explain why the number of patients undergoing postoperative antispasmodic exceeded than those in the preoperative [20].

Patients initiating antispasmodics over a year after surgery were shown to undergo treatment with a shorter duration, as shown in Fig 2. A possible explanation is that patients initiating antispasmodics within a year postoperatively were more likely to be influenced by preoperative and perioperative factors. However, patients initiating antispasmodics over a year after surgery were more likely to be associated with new-onset LUTS symptoms. Patients in this group tend to suffer from poor compliance to antispasmodics due to its side effects, and thus presented with a shorter medication duration [30]. On the contrary, patients who initiated adrenergic alpha-blockers and antispasmodics within 2–6 months postoperatively tend to undergo medical treatments longer. Relatively short duration of postoperative adrenergic alpha-blocker usage in patients starting at 6–12 months after surgery was noted. This may be biased by relatively fewer patients in this group, which only accounted for 7.41% of all patients receiving postoperative adrenergic alpha-blockers. In addition, the follow-up time of postoperative medications usage may also be confined and biased by the relatively short follow-up time of our study, which a median of 5.3 years was obtained.

This study discloses the factors associated with the medications usage after operation for patients with BPH. However, there are some limitations. Firstly, some data were unable to obtain due to the retrospective nature of this study such as IPSS. Postoperative urodynamic tests and cystoscopy were not regularly performed in these patients. This may result in the lack of objective measurements of symptom improvement after surgery. Furthermore, urethral stricture related to surgery may not be completely identified. However, these patients were followed up in VGHTC, and further examinations may be performed after evaluation of urologists.

Secondly, the severity and the duration of the comorbidities were not included in this study. In fact, longer diabetes duration was associated with OAB in diabetic patients [31]. In addition, patients with CKD progressed to end stage renal disease often present with OAB symptoms [32]. Further classifying comorbidities based on severity and duration may shed more light on future studies. Thirdly, some of the symptomatic patients may refuse medications after surgery, while some patients with minimal residual symptoms may insist on medications. The patient preference of medical treatments may result in bias in this study.

Perioperative factors may influence the outcome of the surgeries, and further affect postoperative medications. These factors include surgical techniques, experience of the surgeon and resected prostate regions. In this study, laser enucleation and photoselective vaporization were categorized in laser intervention. These two techniques present with similar surgical outcome and functional outcome [33,34]. However, the difference in the technique selection may still lead to bias in medication usage.

Patients may receive medical treatment from other hospitals, where the medical records were not included in this study. This may result in bias that we did not record all the medical treatments these patients underwent. To deal with this problem, data collection through Taiwan’s National Health Insurance Research Database may provide more thorough medical records for further research.

## Conclusion

BPH patients with CKD are more likely to undergo adrenergic alpha-blockers after prostate surgery. In the meantime, patients who underwent antispasmodic medications before surgery are more liable to receive antispasmodics postoperatively. In addition, patients who received greater resected prostate weight to preoperative prostate volume ratio are less likely to undergo antispasmodic medications after surgery. For patients who undergo medications after prostate surgery, most of them started medications within a year postoperatively.

## Supporting information

S1 File (XLSX)
